# Risk factors associated with lower defecation frequency in hospitalized older adults: a case control study

**DOI:** 10.1186/s12877-015-0041-0

**Published:** 2015-04-10

**Authors:** Jen-Tzer Gau, Utkarsh H Acharya, M Salman Khan, Tzu-Cheg Kao

**Affiliations:** Department of Geriatric Medicine/Gerontology, Ohio University Heritage College of Osteopathic Medicine, Athens, OH 45701 USA; Division of Hematology-Oncology, Department of Internal Medicine, University of Arizona Cancer Center, Tucson, AZ 85724 USA; Division of Pulmonary, Critical Care, Sleep Medicine & Allergy, University of Nebraska Medical Center, Omaha, NE 68198 USA; Division of Epidemiology and Biostatistics, Department of Preventive Medicine and Biometrics, Uniformed Services University of the Health Sciences, Bethesda, MD 20814 USA

**Keywords:** Defecation, Bowel movement, Hospitalized elderly, Acute exacerbation of COPD

## Abstract

**Background:**

Constipation is highly prevalent in older adults and may be associated with greater frequency of acute exacerbation of chronic obstructive pulmonary disease (AECOPD). We investigated the prevalence of lower defecation frequency (DF) and risk factors (including AECOPD) associated with lower DF among hospitalized elderly patients.

**Methods:**

We conducted a retrospective case–control study in a community hospital of Southeast Ohio. Adults aged 65 years or older admitted during 2004 and 2006 were reviewed (N = 1288). Patients were excluded (N = 212) if their length of stay was less than 3 days, discharge diagnosis of *Clostridium difficile*-associated diarrhea, death or ventilator- dependent respiratory failure during hospitalization. Lower DF was defined as either an average DF of 0.33 or less per day or no defecation in the first three days of hospitalization; cases (N = 406) and controls (N = 670) were included for the final analysis.

**Results:**

Approximately 38% had lower DF in this patient population. Fecal soiling/smearing of at least two episodes was documented in 7% of the patients. With the adjustment of confounders, AECOPD (adjusted odds ratio [AOR] =1.47, 95% confidence interval [CI] =1.01-2.13) and muscle relaxant use (AOR =2.94; 95% CI =1.29-6.69) were significantly associated with lower DF. Supplementation of potassium and antibiotic use prior to hospitalization was associated with lower risk of lower DF.

**Conclusions:**

Approximately 38% of hospitalized older adults had lower DF. AECOPD and use of muscle relaxant were significantly associated with lower DF; while supplementation of potassium and antibiotic use were protective for lower DF risk after adjusting for other variables.

**Electronic supplementary material:**

The online version of this article (doi:10.1186/s12877-015-0041-0) contains supplementary material, which is available to authorized users.

## Background

Constipation is highly prevalent in older adults [1-3]. It was estimated that up to 55% of nursing home residents received laxatives regularly [3], and 42% of patients admitted to two acute geriatric wards had fecal impaction [4]. While the majority of constipated patients have a benign clinical course with their constipation being considered functional in nature, chronic constipation has been associated with poor quality of life and reduced survival [5].

There is evidence that gastrointestinal conditions such as constipation [6] and gastro-esophageal reflux disease [7] may be associated with acute exacerbations of chronic obstructive pulmonary disease (AECOPD). It was shown that 40% of patients with stable COPD reported constipation and abdominal distention symptoms [6]. As COPD is highly prevalent and the third leading cause of death among older adults in the USA [8], its possible association with constipation raises a clinical need to explore efficacious prevention and therapeutic strategies to improve its clinical outcome.

Despite the high prevalence of constipation, little information is available on the patterns of bowel health and defecation frequency (DF) among hospitalized adults. The purpose of this study was to report the prevalence of lower DF and to analyze associated risk factors (including AECOPD) with lower DF of hospitalized elderly patients in a community hospital.

## Methods

### Patients and data

Data were retrieved from a local community hospital of Southeast Ohio. This dataset was previously described elsewhere [9]. Briefly, adults aged 65 years or older admitted during 2004 and 2006 were reviewed (N = 1288). Only the first admission was reviewed for patients with readmission(s). Patients were excluded (N = 212) if their hospital stay was less than 3 days, stool was positive for *Clostridium difficile* toxin, hospitalization-related mortality, on ventilator, or records were incomplete. Written informed consent for participation in the study was not obtained (and not required) from participants. This study was approved by the Ohio University Institutional Review Board.

Data included the number of defecation during hospitalization as documented by nursing staff, demographics, smoking status, medical history, medication use, admission/discharge diagnoses, and laboratory results (serum albumin, calcium, and potassium levels – obtained from the first set of blood). Medication use prior to admission was recorded as current use vs. non-use. The diagnosis of AECOPD was based on clinical diagnosis with the confirmation of chest x-rays to avoid the overlapped diagnosis with pneumonia [9]. The diagnosis of acute congestive heart failure (CHF), pneumonia, and AECOPD were discharge diagnoses with which patients were mainly treated for during the hospital stay.

### Definition of cases

It is commonly agreed that fewer than three defecations per week is considered infrequent bowel movement and constitutes one element of the diagnostic criteria for functional constipation [10]. Given the fact that not all patients stayed in the hospital for more than one week, we decided to use the average DF per day (as calculated by the number of total defecations divided by the number of hospitalization days) as an indicator of lower DF. Those with an average DF 0.33 or less per day (i.e., ≤ one defecation in three days) or having no defecation for the first three days of hospitalization were considered lower DF (Cases; N = 406); those without the above conditions were grouped as the controls (N = 670). Episodes of fecal soiling/smearing were also recorded and not considered as defecation.

### Missing data

Were encountered in the smoking history and laboratory results. Patients without documented data were excluded from data analysis.

### Statistical analysis

Mean, standard deviation (SD), and percentage (%) with frequency were used to report continuous and discrete variables. Chi-square test and two-sample t-tests (two-sided) were used to assess if there was a significant association between two groups. Multiple logistic regression with odds ratio (OR) with a 95% confidence interval (CI) was used to measure the association between a risk factor and lower DF after adjusting for other variables in the model.

Potential confounders used in the multiple logistic regression model analysis included age, gender (Model 1), pneumonia, and acute congestive heart failure (Model 2), current smoking status, cognitive impairment, supplementation of potassium, calcium, and iron, anti-cholinergic drugs (including antipsychotics, H1 antihistamines, antimuscarinic receptor blocker, and antispasmodic drugs), diuretics, narcotics, muscle relaxants, and oral antibiotic use prior to admission (Model 3), and uses of β2 agonists and anti-cholinergic bronchodilators (Model 4, shown in Additional file 1).

Hosmer and Lemeshow goodness-of-fit test was used to assess the fit of the model to the data. Regression diagnostics were used to assess if a model is reasonable. Statistical significance was set at a level of .05. Statistical software package, PC SAS version 9.3 (SAS Institute, Inc., Cary, NC) was used to perform the statistical analyses.

## Results

Approximately 26% (281/1076) of patients had an average DF of 0.33 or less per day, and 26% (284/1076) had no defecation for the first three days of hospitalization; 38% (406/1076) had either one of the above. Fecal soiling/smearing of at least two episodes was documented in 7% (74/1076) of the study patients.

Both case and control groups were similar in the mean age (mean ± SD, 79.0 ± 8.2 vs. 79.9 ± 8.3 years), female gender (64% vs. 66%), current smoking status (13% vs. 11%), and mean blood levels of potassium, calcium and albumin on admission (all p values ≥0.10; Table 1). The case group was taking muscle relaxants more often (4% vs. 2%), but significantly less in the supplementation of potassium, calcium, and iron as well as the antibiotics prior to admission than the control group. Both case and control groups were similar in the use of anti-cholinergic drugs, calcium channel blockers, proton pump inhibitors, narcotics, and anti-cholinergic and β2 agonist bronchodilators (Table 1).

**Table 1:** **Characteristics of cases with lower defecation frequency (DF)** ^**a**^ **and controls among hospitalized older adults**

**Variables** ^**b**^	**Cases (N = 406)**	**Controls (N = 670)**	**P value**
Mean age (yr) ± SD	79.0 ± 8.2	79.9 ± 8.3	0.104
Female	260 (64%)	440 (66%)	0.586
White	404 (99.5%)	663 (99%)	NA
Admitted from nursing homes	60 (15%)	127 (19%)	0.080
Ex-smoker	94 (23%)	137/667^c^ (21%)	0.313
Current smoker	53 (13%)	70/666^c^ (11%)	0.205
**Medical History**			
CAD	139 (34%)	217 (32%)	0.532
COPD	122 (30%)	215 (32%)	0.484
Diabetes	130 (32%)	197 (29%)	0.366
CHF	83 (20%)	172 (26%)	0.051
Depression	95 (23%)	182 (27%)	0.171
Stroke	58 (14%)	108 (16%)	0.420
Cognitive impairment	51 (13%)	114 (17%)	0.05
**Medications**			
Muscle relaxant	17 (4%)	11 (2%)	0.016‡^d^
Anti-cholinergic drugs^e^	88 (22%)	180 (27%)	0.056
Calcium channel blocker	87 (21%)	129 (19%)	0.338
Potassium supplement	74 (18%)	189 (28%)	<0.001‡
Calcium supplement	64 (16%)	143 (21%)	0.024‡
Iron supplement	26 (6%)	79 (12%)	0.004‡
Oral laxatives^f^	44/261 (17%)	111/458 (24%)	0.021‡
Antibiotic use prior to Admission	32 (8%)	102 (15%)	<0.001‡
Proton pump inhibitor	152 (37%)	266 (40%)	0.460
Diuretics	178 (44%)	336 (50%)	0.045‡
Narcotics	106 (26%)	175 (26%)	0.999
NSAIDS	52 (13%)	73 (11%)	0.343
Anti-cholinergic	51 (13%)	90 (13%)	0.681
Bronchodilators			
β2 agonists inhalers	81 (20%)	149 (22%)	0.375
Steroid inhalers	51 (13%)	73 (11%)	0.407
**Acute diagnosis** ^g^ **of hospital stay**			
Pneumonia	81 (20%)	126 (19%)	0.644
AECOPD	66 (16%)	81 (12%)	0.054
Acute CHF	52 (13%)	81 (12%)	0.729
**Admission blood tests**			
Potassium (mmol/L)	4.1 ± 0.6 (N = 405)	4.1 ± 0.6 (N = 668)	0.993
Calcium (mg/dL)	9.1 ± 0.6 (N = 400)	9.1 ± 0.6 (N = 665)	0.520
Albumin (gm/dL)	3.2 ± 0.53 (N = 343)	3.2 ± 0.52 (N = 600)	0.416

^a^Patients with less than 3 days of hospital stay were excluded. Lower DF was defined as having either an average DF ≤ 1/3 per day or no defecation in the first 3 days during hospital stay.

^b^Data are presented as number (%) or mean ± SD unless indicated otherwise.

^c^Information on ex-smoke and current smoking status was not documented in three and four patients, respectively, of the control group.

^d^Calculated by Fisher’s exact test.

^e^Anticholinergic drugs included antipsychotics, H1 antihistamines, antimuscarinic receptor blocker, and antispasmodic drugs.

^f^Information on oral laxative use was only available in 261 of 406 (64%) patients in the case group, and in 458 of 670 (68%) patients in the control group.

^g^Only the three major diagnoses were listed.

‡ Significant (p < .05).

*Abbreviations:* *CAD* coronary artery disease, *CHF* congestive heart failure, *COPD* chronic obstructive pulmonary disease, *AECOPD* Acute exacerbation of COPD, *NA* not applicable, *NSAIDs* non-steroidal anti-inflammatory drugs, *SD* standard deviation.

With the adjustment of age and gender (Model 1), and acute diagnosis of pneumonia and congestive heart failure (Model 2), AECOPD was not associated with lower DF (results shown in Additional file 1). With further adjustment of current smoking status, cognitive impairment, and supplementation as well as medication uses (Model 3), AECOPD (adjusted OR = 1.47, 95% CI = 1.01-2.13) and uses of muscle relaxants (AOR =2.94; 95% CI =1.29-6.69) were significantly associated with lower DF by the multiple logistic regression model (Table 2 and Additional file 1). On the other hand, supplementation of potassium and iron, and antibiotic use prior to hospitalization were significantly associated with lower risk of lower DF. The model was reasonable as regression diagnostics showed no collinearity among risk factors; no noticeable outlier or influential observations; and the model fit data very well (Chi-square =6.77, df = 8, p = 0.56, Hosmer and Lemeshow goodness-of-fit test). Similar results held if we further adjusted for uses of β2 agonists and anti-cholinergic bronchodilators (model 4 in Additional file 1).

**Table 2:** **Odds ratio for the risk factors associated with lower defecation frequency (DF) among hospitalized older adults in the multiple logistic regression model**

**Variables** ^**a**^	**Case [N = 406] (Yes/No)**	**Control [N = 670] (Yes/No)**	**Crude OR [95% CI]**	**Adjusted OR [95% CI] (406 cases, 666 controls)**
Age (in years)	--	--	0.99 [0.97, 1.00]	1.0 [0.98, 1.02]
Gender (female = 1; male = 0)	260/146	440/230	0.93 [0.72, 1.20]	1.05 [0.80, 1.38]
*AECOPD*	*66/340*	*81/589*	*1.41 [0.99, 2.01]*	*1.47 [1.01, 2.13]* ^*c*^
Pneumonia	81/325	126/544	1.08 [0.79, 1.47]	1.29 [0.92, 1.80]
Acute CHF	52/354	81/589	1.07 [0.74, 1.55]	1.22 [0.82, 1.81]
Current smoking	53/353	70/596	1.28 [0.87, 1.87]	1.08 [0.72, 1.62]
History of cognitive impairment	51/355	114/556	0.70 [0.49, 1.00]	0.82 [0.56, 1.21]
**Medication uses**				
Muscle relaxants	17/389	11/659	2.62 [1.21, 5.65]	2.94 [1.29, 6.69]^c^
Anti-cholinergic drugs^*b*^	88/318	180/490	0.75 [0.56, 1.01]	0.78 [0.58, 1.07]
Potassium supplement	74/332	189/481	0.57 [0.42, 0.77]	0.64 [0.46, 0.89]^c^
Calcium supplement	64/342	143/527	0.69 [0.50, 0.95]	0.76 [0.54, 1.07]
Iron supplement	26/380	79/591	0.51 [0.32, 0.81]	0.57 [0.35, 0.92]^c^
Diuretics	178/228	336/334	0.78 [0.61, 0.99]	0.87 [0.67, 1.14]
Narcotics	106/300	175/495	1.00 [0.75, 1.32]	1.07 [0.79, 1.44]
Antibiotics	32/374	102/568	0.48 [0.31, 0.72]	0.45 [0.29, 0.69]^c^

^a^Variables were dichotomized as 1 = yes, 0 = no (baseline) unless stated otherwise. The adjusted OR with a 95% CI for the association between a risk factor and the presence of lower DF was estimated after adjusting for all other variables listed in this Table.

^b^Anticholinergic drugs include antipsychotics, H1 antihistamines, anti-muscarinic receptor blocker, and antispasmodic drugs.

^c^Significant (p < 0.05).

Regression diagnostics showed no collinearity among risk factors, no noticeable outlier, no influential or ill-fitted observations, and the model fit data very well (Chi-square =6.77, df = 8, p = 0.56, Hosmer and Lemeshow goodness-of-fit test).

*Abbreviations:* *AECOPD* acute exacerbation of chronic obstructive pulmonary disease, *CHF* congestive heart failure, *CI* confidence interval, *OR* odds ratio.

## Discussion

Our study demonstrated that approximately 38% of hospitalized older adults had lower DF during their hospital stay. The risk factors that were associated with lower DF were the use of muscle relaxants and those with AECOPD after adjusting for confounders. On the other hand, supplementation of potassium and iron and antibiotic use were associated with a lower risk for lower DF.

This study found that AECOPD was associated with higher risk of lower DF during the hospital stay after adjusting for confounders that were listed in Table 2. The unadjusted OR for the association was 1.41 [95% CI = 0.99 - 2.01], which was very close to the significant level set at 0.05. As many conditions could alter defecation frequency, we adjusted for confounders in the multiple logistic regression models by including those risk factors that are known associated with constipation (e.g., supplementation of calcium, medications with anticholinergic effects, and narcotic drugs) as well as those that were significantly different between the case and control groups in this study (e.g., cognitive impairment, and supplementation of potassium and iron). With the adjustment of the above risk factors, patients with AECOPD had approximately 47% higher risk for lower DF (shown in Table 2). It is possible that the association may be further confounded by the anti-cholinergic side effects of bronchodilators which were commonly used during hospitalizations. With the inclusion of additional confounders for COPD (i.e., uses of β2 agonists and anti-cholinergic bronchodilators) into the model (model 4, results shown in Additional file 1), the association between lower DF and AECOPD remained significant (adjusted OR = 1.50, 95% CI = 1.01 - 2.22). Therefore, it was less likely that bronchodilator use accounted for the association.

Several clinical trials have shown that maintenance treatment with azithromycin (a macrolide) significantly decreased the exacerbation rate of COPD and improved quality of life [11,12]. In addition to the immunomodulatory, anti-inflammatory, and antibacterial effects of azithromycin, it is also known that macrolides stimulate gastrointestinal motility [13] which may present as diarrhea [12]. One of our speculations is that the benefit from azithromycin treatment on the reduction of COPD exacerbation rate may be exerted on the improvement of bowel motility and function in addition to its antibacterial effects. The clinical implication from our study may suggest that improving bowel motility (as shown by higher DF) may decrease the risk of AECOPD. It would require further studies to test the above hypothesis and the potential mechanism(s) that may explain the association between lower DF and AECOPD.

Medication’s side effects as the cause of constipation are also commonly encountered in the elderly population. Our study demonstrated that use of muscle relaxants was associated with lower DF, which was consistent with other reported findings [3,14,15]. Ueki et al. further extended this finding to the use of hypnotics conjecturing that mechanism of constipation may be induced by the myorelaxant effect of these medications during hospitalization [15].

Alternatively, our study revealed that use of potassium supplement and oral antibiotics prior to admission posed a lower risk for impaired DF. This was consistent with our current knowledge as potassium supplements improve bowel motility in the conditions of potassium deficiency [16] and may be associated with gastric irritation [17] which may increase bowel motility. The effect of oral antibiotics (particularly macrolide and penicillin) in increasing bowel motility (which may lead to diarrhea) is well known. In contrast, iron supplementation demonstrated a protective benefit for lower DF in our study. While iron supplement has been characteristically associated with chronic constipation [14], iron uses may also cause upset stomach, nausea, and diarrhea as side effects.

Although lower DF itself may not imply constipation status, infrequent bowel movement (i.e., fewer than three defecations per week) is one of the features defining constipation. Due to the lack of comprehensive subjective data, the case group may not have captured those patients who had normal DF but clinically felt constipated (i.e., straining, having lumpy or hard stool). Despite the limitation, DF was an objective feature that was readily available in our patients’ record for data analysis.

There were several limitations in our study. First, documentation of defecations depended on the reports from both nursing staff and patients. Second, patient-centered, subjective descriptions of constipation during the hospital stay were not available. Data of constipation history and laxative use were not readily available and thus, were not included for adjustment of confounders in the multiple logistic regression modeling. Furthermore, constipated patients often take over-the-counter laxatives for self-treatment, and it was not possible in capturing all the above information in a retrospective study. Third, potential confounders associated with lower DF including physical activity, functional status, and new medication use during the hospital stay were not available for data analysis. Lastly, the study patient population was mainly white and constituents of a single study site which precluded one from making generalizable conclusions.

## Conclusions

This retrospective case–control study found that 38% of hospitalized older adults had lower DF and up to 7% of patients had fecal smearing during hospital stay in a rural community. AECOPD and use of muscle relaxants were significantly associated with lower DF; whereas supplementation of potassium and antibiotic use prior to hospitalization was protective for lower DF risk after adjusting for other confounders. Future studies focusing on the association between improving bowel health and reduction of COPD exacerbation may assist in forming more concrete hypothesis relating to how these two clinical entities inter-relate to one another.

## Additional file

Additional file 1:
**Association between lower defecation frequency (DF) and acute exacerbation of COPD among hospitalized older adults.**

## Abbreviations

AECOPD: Acute exacerbation of chronic obstructive pulmonary disease
CAD: Coronary artery disease
CHF: Congestive heart failure
CI: Confidence interval
COPD: Chronic obstructive pulmonary disease
DF: Defecation frequency
NA: Not applicable
NSAIDs: Non-steroidal anti-inflammatory drugs
OR: Odds ratio
SD: Standard deviation
