# Supplementary material for: Risk factors associated with lower defecation frequency in hospitalized older adults: a case control study
Source: BMC Geriatr. 2015 Apr 10;15:44. doi: 10.1186/s12877-015-0041-0 (PMC4397888; doi:10.1186/s12877-015-0041-0)
Supplement: Additional file 1: — Association between lower defecation frequency (DF) and acute exacerbation of COPD among hospitalized older adults. [file 12877_2015_41_MOESM1_ESM.doc]

| **Appendix Table. Association between lower defecation frequency (DF) and acute exacerbation of COPD among hospitalized older adults** | | | | | | | |
| --- | --- | --- | --- | --- | --- | --- | --- |
|  | **Lower DF Case (N=406] (Yes/No)** | **Control [N=670]**  **(Yes/No)** | **Crude OR [95% CI]** | **Adjusted OR**  **[95% CI]**  **Model 1**^a^ | **Adjusted OR**  **[95% CI]**  **Model 2**^b^ | **Adjusted OR**  **[95% CI]**  **Model 3**^c^ | **Adjusted OR**  **[95% CI]**  **Model 4**^d^ |
| **AECOPD** | 66/340 | 81/589 | 1.41 [0.99, 2.01] | 1.35 [0.95, 1.93] | 1.34 [0.94, 1.92] | 1.47 [1.01, 2.13] | 1.50 [1.01, 2.22] |

^a^ Model 1 was adjusted for age and gender.

^b^ Model 2 was adjusted for age, gender, pneumonia, and acute congestive heart failure.

^c^ Model 3 was adjusted for all the variables included in the Model 2 plus the following: current smoking status, cognitive impairment, supplementation of potassium, calcium, and iron, anti-cholinergic drugs (including antipsychotics, H1 antihistamines, antimuscarinic receptor blocker, and antispasmodic drugs), diuretics, narcotics, muscle relaxants, and oral antibiotic use prior to admission.

^d^ Model 4 was adjusted for all the variables included in the Model 3 plus the following: uses of β2 agonists and anti-cholinergic bronchodilators. Regression diagnostics for Model 4 showed no collinearity among risk factors, no noticeable outlier, no influential or ill-fitted observations, and the model fit data well (Chi-square =9.61, df=8, p=0.29, Hosmer and Lemeshow goodness-of-fit test).

**Abbreviations**: AECOPD= acute exacerbation of chronic obstructive pulmonary disease; CI= confidence interval; OR= odds ratio.
